# Consumption of *Buglossoides arvensis* seed oil is safe and increases tissue long-chain *n*-3 fatty acid content more than flax seed oil – results of a phase I randomised clinical trial

**DOI:** 10.1017/jns.2015.34

**Published:** 2016-01-08

**Authors:** Natalie Lefort, Rémi LeBlanc, Marie-Andrée Giroux, Marc E. Surette

**Affiliations:** 1 Department of Chemistry and Biochemistry, Université de Moncton, Moncton, NB, Canada; 2 Réseau de Santé Vitalité Health Network, Centre hospitalier universitaire Dr-Georges-L.-Dumont, Moncton, NB, Canada; 3 Department of Biology, Université de Moncton, Moncton, NB, Canada; 4 Department of Biology, Université du Québec à Rimouski, Rimouski, QC, Canada

**Keywords:** Stearidonic acid, Leucocytes, EPA, AE, adverse event, ALA, α-linolenic acid, AR, adverse reaction, DPA, docosapentaenoic acid, DGLA, dihomo-γ-linolenic acid, ETA, eicosatetraenoic acid, FAME, fatty acid methyl esters, GLA, γ-linolenic acid, HBSS, Hanks' balanced salt solution, PMN, polymorphonuclear cells, SDA, stearidonic acid

## Abstract

Enrichment of tissues with ≥20-carbon *n*-3 PUFA like EPA is associated with positive cardiovascular outcomes. Stearidonic acid (SDA; 18 : 4*n*-3) and α-linolenic acid (ALA; 18 : 3*n*-3) are plant-derived dietary *n*-3 PUFA; however, direct comparisons of their impact on tissue *n*-3 PUFA content are lacking. Ahiflower^®^ oil extracted from *Buglossoides arvensis* seeds is the richest known non-genetically modified source of dietary SDA. To investigate the safety and efficacy of dietary Ahiflower oil, a parallel-group, randomised, double-blind, comparator-controlled phase I clinical trial was performed. Diets of healthy subjects (*n* 40) were supplemented for 28 d with 9·1 g/d of Ahiflower (46 % ALA, 20 % SDA) or flax seed oil (59 % ALA). Blood and urine chemistries, blood lipid profiles, hepatic and renal function tests and haematology were measured as safety parameters. The fatty acid composition of fasting plasma, erythrocytes, polymorphonuclear cells and mononuclear cells were measured at baseline and after 14 and 28 d of supplementation. No clinically significant changes in safety parameters were measured in either group. Tissue ALA and EPA content increased in both groups compared with baseline, but EPA accrual in plasma and in all cell types was greater in the Ahiflower group (time × treatment interactions, *P* ≤ 0·01). Plasma and mononuclear cell eicosatetraenoic acid (20 : 4*n*-3) and docosapentaenoic acid (22 : 5*n*-3) content also increased significantly in the Ahiflower group compared with the flax group. In conclusion, the consumption of Ahiflower oil is safe and is more effective for the enrichment of tissues with 20- and 22-carbon *n*-3 PUFA than flax seed oil.

*n*-3 PUFA are nutrients that are naturally enriched in common foods such as fatty fish and several plant-derived oils like rapeseed and soyabean oils. Dietary supplements such as fish oils and flax seed oil are also rich sources of *n*-3 PUFA while other foods including eggs and baked goods that have been supplemented with *n*-3 PUFA are increasingly available^(^^1^^)^. While a review of more recent clinical reports has questioned the health benefits of consuming *n*-3 PUFA supplements^(^^2^^)^, there is a more extensive literature spanning several decades indicating that dietary *n*-3 PUFA promote health and prevent disease^(^^3^^–^^9^^)^.

The simplest *n*-3 PUFA is α-linolenic acid (ALA; 18 : 3*n*-3) and is commonly found in plant-derived dietary oils such as flax, rapeseed and soyabean oils (Fig. 1). The other common dietary *n*-3 PUFA are the longer-chain and more unsaturated PUFA that are usually associated with fish and fish oils. These fatty acids include the 20-carbon EPA (20 : 5*n*-3) and the 22-carbon docosapentaenoic acid (DPA; 22 : 5*n*-3) and DHA (22 : 6*n*-3). Following their consumption, these longer-chain *n*-3 PUFA are preferentially incorporated into cellular membrane phospholipids where they participate in the proper functioning of membrane lipid bilayers and are precursors of pro-resolving anti-inflammatory lipid mediators^(^^10^^)^.

**Fig. 1.**
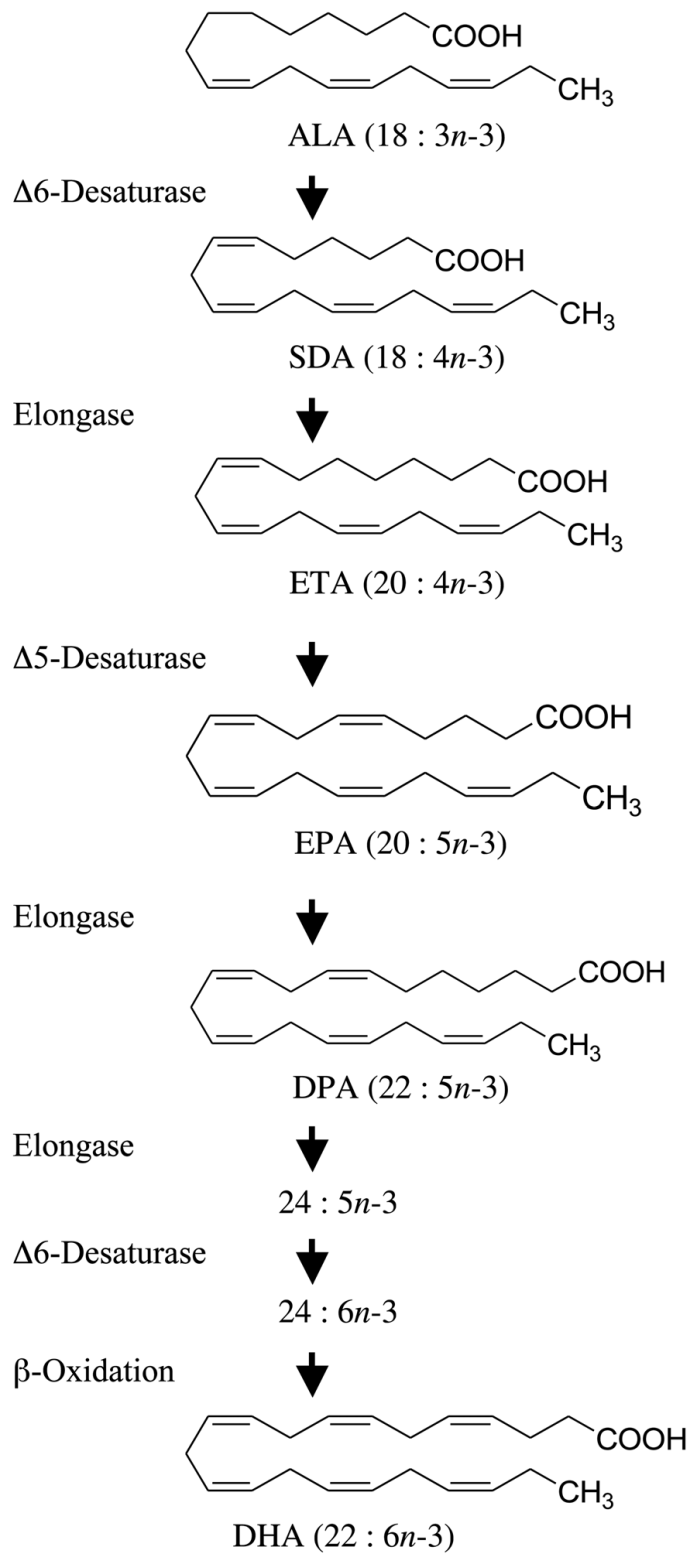
Metabolic *n*-3 PUFA pathway showing the enzymic conversion of α-linolenic acid (ALA) to DHA. Enzymes responsible for each metabolic step are shown on the left. SDA, stearidonic acid; ETA, eicosatetraenoic acid; DPA, docosapentaenoic acid.

The *n*-3 PUFA found in fish are minor dietary constituents in populations consuming Western diets^(^^1^^,^^11^^)^ and are consumed in amounts that are inferior to recommended intakes^(^^1^^,^^3^^,^^12^^)^. However, the demand for these fatty acids has increased over the last decade while global fish oil supplies have diminished and projections indicate that demand could soon outpace supply^(^^13^^–^^15^^)^. Alternative sources of *n*-3 PUFA have been sought to meet this demand, not only for human consumption but also as a feed ingredient in aquaculture, which commands a significant share of the global fish oil production^(^^14^^)^.

Perhaps the most obvious alternative to fish oils that can be produced in a sustainable manner is plant-derived oils. A number of plants produce seed oils that are enriched with ALA such as flax (approximately 50 % ALA), soya (7 % ALA) and rapeseed (9 % ALA). Although humans express the enzymes necessary for the conversion of dietary ALA to any of the other members of the *n*-3 family of PUFA (Fig. 1), in populations consuming typical Western diets the consumption of foods or supplements containing ALA results in limited accumulation of EPA, DPA or DHA in cells and tissues^(^^16^^–^^20^^)^. Since the beneficial health effects associated with the consumption of EPA- and DHA-rich foods are largely associated with changes in tissue membrane fatty acid composition, most of the health benefits attributed to the consumption of fish and fish oils are not attained with the consumption of vegetable oils containing ALA. Although some beneficial biological activity has been associated with the consumption of ALA, the associated health benefits are not as well established^(^^20^^–^^24^^)^, and health benefits would be largely independent of its conversion to longer-chain *n*-3 PUFA.

A plant-derived *n*-3 PUFA that has generated recent interest is stearidonic acid (SDA; 18 : 4*n*-3)^(^^25^^)^. SDA is found in the seeds of a number of plant species from the Boraginaceae family^(^^26^^,^^27^^)^. This 18-carbon *n*-3 PUFA is the immediate product following the conversion of ALA catalysed by Δ-6 desaturase (Fig. 1). Stable isotope tracer studies have shown that this metabolic step is not efficient in humans^(^^16^^)^ and is the reason why consumption of ALA results in little tissue accumulation of longer-chain more unsaturated *n*-3 PUFA^(^^16^^–^^20^^)^. Because SDA metabolism bypasses this rate-limiting step in the pathway, the consumption of oils rich in SDA can result in an enrichment of tissues with longer-chain PUFA like EPA and DPA. However, an impact on tissue DHA is unlikely because of a second Δ-6 desaturase-catalysed step required for DHA synthesis.

Few seed oils containing SDA have been produced for human consumption. *Ribes nigrum* seed oil (blackcurrant oil; 2–4 % SDA) and *Echium plantagineum* seed oil (echium oil; 12–14 % SDA) are produced commercially, and rapeseed and soyabeans^(^^28^^,^^29^^)^ have been genetically modified to produce seeds enriched in SDA (SDA omega-3 soybeans; 20–30 % SDA)^(^^30^^)^. A number of clinical trials have investigated the impact of dietary SDA-ethyl ester, echium oil or SDA soybean oil on tissue fatty acid composition and have shown that tissue EPA content, but not DHA, is significantly elevated following the consumption of dietary SDA^(^^18^^,^^31^^–^^41^^)^. While some studies report the consumption of SDA oils and ALA oils^(^^18^^,^^38^^)^, no clinical trials have directly compared the efficacy of SDA-containing oil with ALA-containing oils for the enrichment of human tissues with long-chain *n*-3 PUFA.

The present study investigates a new plant oil extracted from the seeds of *Buglossoides arvensis* (Ahiflower oil^®^), a rich natural source of SDA (20 % SDA). This 28-d single-site, parallel-group, randomised, double-blind, comparator-controlled phase I clinical trial is the first to study the consumption of *Buglossoides* oil in humans. In addition to measuring safety parameters, this is also the first trial in humans in which the capacity of dietary SDA to enrich tissues with long-chain *n*-3 PUFA is statistically compared with that of dietary ALA, which was supplied as flax seed oil.

## Subjects and methods

### Study approval and ethics

Approval of the investigational dietary oils (Ahiflower and flax seed oils) and the clinical trial design was obtained from the Natural Health Product Directorate of Health Canada (HC-NHPD-196699). This study was conducted according to the guidelines laid down in the Declaration of Helsinki and all procedures involving human subjects were approved by the ethics committees for human research of the Réseau de Santé Vitalité Health Network and of the Université de Moncton. Written informed consent was obtained from all subjects. This study is registered with clinicaltrials.gov (identifier: NCT02226354).

### Study dietary oils

Oils from flax (*Linum usitatissimum*) seeds and from Ahiflower (*B. arvensis*) seeds were provided by Technology Crops International. Antioxidant (Fortium RPT 40 IP Rosemary extract + ascorbyl palmitate; Kemin Industries) and lemon flavour (FONA International) were added, yielding 9·73 ml of refined oil per dosage unit of 10 ml. Detailed fatty acid profiles of the formulations are shown in Table 1.

**Table 1.** Fatty acid profile of Ahiflower and flax seed oils (% total fatty acids)*

Fatty acid	Ahiflower	Flax seed
16 : 0	5·2	5·3
16 : 1*n*-7	0·1	ND
18 : 0	1·9	2·8
18 : 1*n*-9	7·5	13·9
18 : 2*n*-6	11·8	17·5
18 : 3*n*-6 (GLA)	5·8	ND
18 : 3*n*-3 (ALA)	46·1	59·1
18 : 4*n*-3 (SDA)	20·1	ND
20 : 0	0·1	ND
20 : 1*n*-11	0·8	ND
22 : 0	0·1	0·1
22 : 1*n*-9	0·1	0·1
24 : 1*n*-9	0·1	0·1
∑ *n*-3 PUFA	66·2	59·1
∑	99·7	98·9

ND, not detected; GLA, γ-linolenic acid; ALA, α-linolenic acid; SDA, stearidonic acid.

* All fatty acids with content of 0·1 % or greater are included. These values are the averages of triplicate measurements.

### Study design

This is a single-centre, randomised, comparator-controlled, parallel-group, double-blind study in healthy subjects. With an expected minimally significant change of 70 % in erythrocyte EPA concentrations^(^^32^^)^, a standard deviation of 50 %^(^^18^^,^^32^^,^^33^^)^, the level of significance set at 0·05 and a desired power of 0·95, the calculated sample size was fifteen subjects per group. In order to account for possible dropouts and non-compliance, twenty subjects were enrolled per group. Recruitment began in May 2014 and was closed in July 2014. The study was completed in August 2014 when twenty participants in each intervention had completed the study. Subjects (18–65 years) responding to recruitment material were asked to answer a pre-screening questionnaire to determine eligibility.

Inclusion criteria included being 18–65 years of age and having a BMI of 18–39·9 kg/m^2^. Exclusion criteria were pregnancy or lactation, medical conditions such as an active peptic ulcer, inflammatory bowel disease, or gastrointestinal bleeding that could influence absorption, metabolism or excretion of the study supplement, the consumption of fish oil or other *n*-3 or *n*-6 PUFA supplements/ drugs within 3 months of beginning the trial, consumption of fatty fish (salmon, herring, mackerel, albacore tuna and sardines) more than twice per month within 3 months of beginning the trial, and unwillingness to avoid PUFA supplements and all fish including shellfish and crustaceans throughout the study period. All inclusion and exclusion criteria can be found in Supplementary Table S1.

**Fig. 2.**
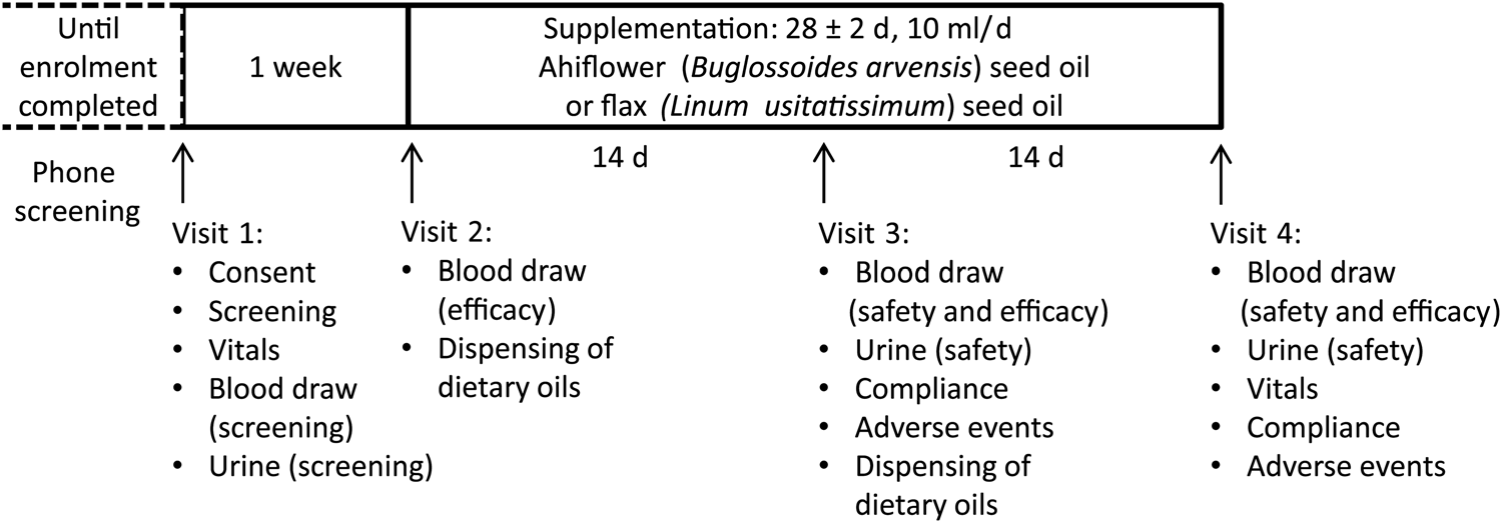
Phase I clinical trial design. Timeline and activities associated with each visit (1–4) are indicated.

Eligible subjects then visited the clinical research team in a reserved conference room in the École de science infirmière at the Université de Moncton (visit 1) at which time they completed a consent form, provided a fasting blood sample and a urine sample, and vital sign measurements were completed (Fig. 2). Following visit 1, subjects were excluded from enrolment in the event of clinically significant abnormal laboratory test results including but not limited to LDL-cholesterol ≥4·1 mm, TAG levels ≥3·95 mm, fasting creatinine ≥15 mg/l, alanine aminotransferase or aspartate aminotransferase ≥1·5 times the upper limit of normal, uncontrolled hypertension (resting systolic blood pressure ≥ 160 mmHg or diastolic blood pressure ≥ 100 mmHg), fasting glucose ≥100 mg/dl (5·55 mmol/l) and HbA1c ≥ 6·0 %. Enrolled subjects were told to maintain their regular dietary and physical activity habits. Subjects were enrolled into the study following review of the screening dossier and written approval by the study physician.

The random allocation sequence, subject enrolment and assignment of subjects to supplementation groups were completed by the study coordinator. Enrolled subjects were randomly assigned to one of two supplementation groups at visit 2, which occurred within approximately 1 week of visit 1. A block randomisation method was adopted; hence a participant enrolment order was generated using an online algorithm (random.org) with two blocks of twenty subjects. The study coordinator, the study nurse, the study physician and the statistician were blinded until all safety and efficacy data were compiled and predetermined statistical analyses completed, at which time the identity of the oil formulations, kept at the bottling facility (Ascenta Health Ltd), was revealed. One group consumed 10 ml/d of the Ahiflower oil formulation (Ahiflower group), the other group consumed 10 ml/d of the flax seed oil formulation (flax group); both groups consumed the oil formulations for 4 weeks. Subjects were given one bottle of oil at visit 2 for days 1–14 and a second bottle at visit 3 for days 15–28. Subjects were instructed to store the bottle of oil in the refrigerator and to consume 10 ml of oil using a single-use oral syringe once per d, preferably at the same time of day, with a meal. They were given the option to consume the oil directly from the syringe or add to a food such as yogurt, pasta sauce or a piece of bread. If they missed a dose, subjects were instructed to take two separate doses the next day, each with a meal. Visit 2 included a blood draw for baseline efficacy endpoint values. Visits 3 and 4 included blood draws and urine tests for the measurement of safety and efficacy endpoints as well as recording of adverse events (AE). Blood and urine samples for the analysis of safety endpoints were promptly forwarded to the Centre Hospitalier Universitaire Dr-Georges-L.-Dumont clinical laboratory in Moncton, NB, for processing.

### Compliance

Adherence to the dosage protocol was verified by measuring residual oil volumes in the bottles returned by the participant. If less than 80 % of the doses over a 2-week period was consumed, the participant was deemed non-compliant and their data not included in the efficacy (fatty acid) analyses.

### Recording of adverse events and reactions

Subjects were informed of their responsibility to report all physical changes (AE) during the study and up to 28 d after the last dose. Open-ended questioning for eliciting AE at all visits was adopted such as ‘how have you felt since your last visit?’ and ‘have you had any new or changed health problems since you were last here?’. Clinical laboratory values more than 1·5 times the upper normal range were also considered to be AE. AE were described in intensity (mild, moderate or severe), in severity (graded using the Common Terminology Criteria for Adverse Events version 4.03)^(^^42^^)^ and causality (unrelated, unlikely, possible, probable, definitely related) by the study physician. AE possibly, probably or definitely related to the study supplement were defined as adverse reactions (AR). The study physician provided any required follow-up to reported AE and AR.

### Blood fractionation for efficacy endpoint measurements

The primary efficacy endpoint was plasma EPA (% of total fatty acids). Secondary efficacy endpoints were plasma eicosatetraenoic acid (ETA; 20 : 4*n*-3) and DPA (% of total fatty acids), and erythrocyte, neutrophil and mononuclear cell ETA, EPA and DPA (% of total fatty acids). Whole blood (20 ml) was collected in Vacutainer tubes on heparin and a 2 ml aliquot was centrifuged (1020 ***g***, 15 min), plasma was collected and centrifuged (3000 ***g***, 20 min) to remove platelets resulting in platelet-free plasma. Pelleted erythrocytes were washed twice by centrifugation (1000 ***g***, 10 min) with 0·9 % saline + 3 mm-Na-EDTA.

Cells from remaining whole blood (18 ml) were collected following dextran sedimentation and centrifugation on a lymphocyte separation medium cushion^(^^43^^)^. Briefly, three volumes of blood were diluted with one volume of Hanks’ balanced salt solution (HBSS) and one volume of 3 % dextran in HBSS and erythrocytes were allowed to settle for 60 min. The remaining cell suspension was diluted with one volume of HBSS and pelleted by centrifugation (200 ***g***, 10 min). Cells were re-suspended in HBSS, placed on a cushion of Lymphocyte Separation Medium (density, 1·077 g/ml) (Wisent) and centrifuged at 900 ***g*** for 20 min at room temperature. The buffy coat containing mononuclear cells was collected from the interface, washed twice and re-suspended in HBSS. Polymorphonuclear cells (PMN) were obtained from the pellet after haemolysis to remove contaminating erythrocytes, were washed and re-suspended in HBSS. Plasma (diluted 1:8 in HBSS) and cell fractions were immediately added to 3·75 volumes of a solution of CHCl_3_–methanol (1:2) and stored at –20°C until lipid extraction.

### Lipid extraction and fatty acid analysis

The internal standard di-heptadecanoyl-PC (Matreya LLC) was added to samples stored in CHCl_3_–methanol. Plasma and cellular lipids were then extracted using the Bligh and Dyer method^(^^44^^)^. The extracts were then saponified with 0·5 m-KOH in methanol (100°C, 15 min). Fatty acid methyl esters (FAME) were prepared by adding 14 % BF_3_ in methanol and heating at 100°C for 10 min^(^^45^^)^. FAME were extracted in hexane and quantified by gas chromatography with flame ionisation detection (GC-FID) using a 30 m BPX-70 column (0·25 mm internal diameter, 0·25 µm film thickness) (SGE Analytical Science) on a Thermo Trace gas chromatograph (Thermo Electron Corporation). The temperature programme was as follows: initial temperature of 150°C with an increase of 10°C/min up to 180°C, followed by an increase of 1·5°C/min until 205°C, with a final increase of 35°C/min until 255°C which was held for 1·9 min. FAME standards (Nu Chek Prep) were used for the determination of FAME peak retention times and for the generation of individual FAME standard curves. The intra-assay precision (% relative standard deviation) of this method for samples containing 50 µg of individual fatty acids per 100 µl plasma was approximately 2 % as previously reported^(^^46^^)^.

### Statistical analyses

A linear model and a generalised linear model were used to identify potential biases in age and sex, respectively, between subjects randomised into either the Ahiflower group or the flax group. Linear models were fitted to determine whether subjects in the Ahiflower and flax groups had similar vital sign measurements (resting heart rate and arterial blood pressure) and weight at baseline and day 28. In each linear model, either heart rate, diastolic pressure, systolic pressure, arterial blood pressure (computed according to Brzezinski^(^^47^^)^) or weight at baseline or day 28 were used as the response, and the variable group (each treatment group) was included as the predictor.

Linear mixed models were fitted to determine whether the percentage of the following fatty acids differed between the Ahiflower and flax groups at baseline, day 14 and day 28 after commencement of supplementation in plasma and circulating cells: ALA, ETA, EPA, DPA and dihomo-γ-linolenic acid (DGLA; 20 : 3*n*-6). The percentage of each fatty acid in each blood fraction was used as the response in each linear mixed model. The variables group (Ahiflower or flax), time, time × treatment interaction, as well as the covariates age, sex and weight were included as predictors, and participant identification number was specified as a random variable. Percentages of DPA in circulating cells were log-transformed to respect assumptions of homoscedasticity and normality of residuals. Because the assumption of homoscedasticity of residuals was not met in the analyses of the percentages of ETA (even after transforming data), linear mixed models were used to analyse the difference in the percentages of ETA between day 28 and baseline (response) as a function of the same set of predictors and the random variable listed above. In these linear mixed models, the heteroscedasticity structure was modelled by specifying a different variance per group (Ahiflower or flax) and per sex^(^^48^^)^. This approach was suitable for most blood fractions, except for the analyses of the percentage of ETA in erythrocytes for which statistical results are not reported, as the heteroscedasticity structure could not be modelled.

Unless otherwise mentioned, all analyses respected the assumptions of normality and homoscedasticity of residuals and were performed with R 3.1.2^(^^49^^)^.

## Results

### Subject characteristics

A total of forty-eight healthy subjects were invited for visit 1, and forty healthy adult women and men were randomised (*n* 20/group). The baseline clinical and anthropometric data of the subjects are shown in Table 2. There were no differences between subjects in the Ahiflower and flax groups with respect to any of the measured baseline parameters.

**Table 2.** Baseline anthropometric and clinical characteristics of enrolled subjects in the Ahiflower and flax groups (Mean values with their standard errors; number of subjects)

	Ahiflower		Flax	
	Mean	sem	Mean	sem
*n*	20		20	
Sex (*n*)				
Female	12		12	
Male	5		7	
Age (years)	30	2	30	2
Weight (kg)	75·4	4	70	4
BMI (kg/m^2^)	26·6	1	25·6	1
Blood pressure (mmHg)				
Systolic	109·1	1·7	110·2	1·6
Diastolic	71·4	1·9	69·8	1·5
Heart rate (beats/min)	69·5	1·6	69·8	2·5
TAG (mmol/l)	1·02	0·08	1·01	0·06
Total cholesterol (mmol/l)	4·38	0·15	4·67	0·19
LDL-cholesterol (mmol/l)	2·43	0·14	2·64	0·17
Non-HDL-cholesterol (mmol/l)	2·88	0·21	3·11	0·19
HDL-cholesterol (mmol/l)	1·49	0·07	1·58	0·09
Glucose (mmol/l)	4·87	0·09	5·14	0·09

### Retention and compliance

In the Ahiflower group, two subjects withdrew from the study; one withdrawal was unrelated to consumption of the dietary oil, whereas the second subject withdrew from the study after craving fatty foods and experiencing acne and insomnia. One participant was removed from all efficacy analyses because of elevated baseline EPA in plasma (1·47 % *v.* mean of 0·46 (sd 0·04) % in the Ahiflower group, significant Grubbs’ test). In the flax group, one participant withdrew after reporting nausea. Two subjects in the flax group were removed from the day 28 analyses due to lack of compliance measured from day 14 to day 28.

### Safety parameters

Subjects in the Ahiflower and flax groups had similar resting heart rate (*P* = 0·9), arterial blood pressure (*P* = 0·8) and weight (*P* = 0·8) at day 28. Fasting blood chemistry, haematology and fasting lipid profiles were assessed at baseline, day 14 and day 28 after the commencement of supplementation and data are presented in Supplementary Table S2. Mean values for each time point showed no clinically significant changes in these safety parameters in either supplement group.

In the Ahiflower group, twenty AE were reported by a total of eight subjects, with six of these AE defined by the study physician as AR, all of which were classified as mild in intensity and of low severity (Supplementary Table S3). In the flax group, twenty-two AE were recorded from a total of ten subjects and of these eight AE were defined by the study physician as AR, all of which were classified as mild in intensity and of low severity.

### Fatty acid analyses

The full fatty acid analyses for each tissue at baseline, day 14 and day 28 are given in the Supplementary Tables S4–S7. Tables 3–6 show the comparisons and statistical analyses of the *n*-3 PUFA profiles between supplement groups in the different tissues.

#### α-Linolenic acid (18 : 3*n*-3)

The ALA content increased in the plasma of subjects from both supplementation groups. In the Ahiflower group, ALA increased (baseline to day 28) from 0·7 to 1·8 % (*P* < 0·001) of total fatty acids. In the flax group, ALA increased from 0·7 to 2·2 % of total fatty acids (*P* < 0·001). After 28 d of supplementation, significant increases from baseline in cellular ALA as a percentage of total fatty acids were measured for both the Ahiflower and flax groups in erythrocytes and PMN where ALA content increased approximately 2-fold, and in mononuclear cells where the increase was approximately 3-fold in both dietary groups. However, there was no difference in the extent of changes in ALA content between the Ahiflower and flax groups in plasma or circulating cells (no time × treatment interaction).

**Table 3.** Plasma *n*-3 PUFA (mol % of total fatty acids) at baseline and following dietary supplementation with Ahiflower or flax seed oils (Mean values with their standard errors)

	Ahiflower group						Flax group						
	Baseline		Day 14		Day 28		Baseline		Day 14		Day 28		
Fatty acid	Mean	sem	Mean	sem	Mean	sem	Mean	sem	Mean	sem	Mean	sem	Time × treatment†: *P*
18 : 3	0·70	0·05	1·50**	0·04	1·82**	0·04	0·72	0·04	1·83**	0·04	2·16**	0·04	NS
18 : 4	0·00	0·00	0·18§	0·00	0·24§	0·02	0·01	0·00	0·01§	0·01	0·00§	0·00	§
20 : 4	0·01	0·00	0·42	0·00	0·41**	0·00	0·03	0·00	0·16	0·00	0·11	0·01	< 0·001‡
20 : 5	0·46	0·01	1·20**	0·03	1·34**	0·00	0·43	0·00	0·78**	0·02	0·76**	0·04	< 0·001
22 : 5	0·58	0·08	0·82**	0·07	0·81**	0·06	0·57	0·07	0·68**	0·10	0·75*	0·15	0·02
22 : 6	1·64	0·05	1·66	0·08	1·62	0·05	1·35	0·07	1·42	0·09	1·33	0·08	ND

ND, not determined.

Mean value was significantly different from that at baseline: * *P* ≤ 0·05, ** *P* ≤ 0·01.

† Ahiflower oil *v.* flax seed oil at 28 d.

‡ This *P* value is not associated with a time × treatment effect, it is rather associated with the effect of oil on the difference between values at baseline and at 28 d.

§ Statistical results are not reported because the heteroscedasticity structure could not be modelled.

**Table 4.** Erythrocyte *n*-3 PUFA (mol % of total fatty acids) at baseline and following dietary supplementation with Ahiflower or flax seed oils (Mean values with their standard errors)

	Ahiflower group						Flax group						
	Baseline		Day 14		Day 28		Baseline		Day 14		Day 28		
Fatty acid	Mean	sem	Mean	sem	Mean	sem	Mean	sem	Mean	sem	Mean	sem	Time × treatment†: *P*
18 : 3	0·15	0·01	0·29**	0·01	0·31**	0·01	0·18	0·01	0·34**	0·01	0·36**	0·01	NS
18 : 4	0·00	0·03	0·01‡	0·04	0·01‡	0·03	0·00	0·03	0·01‡	0·04	0·00‡	0·03	‡
20 : 4	0·02	0·04	0·19‡	0·05	0·21‡	0·03	0·04	0·05	0·10‡	0·05	0·09‡	0·02	‡
20 : 5	0·45	0·01	0·67**	0·02	0·74**	0·01	0·42	0·01	0·52**	0·01	0·55**	0·02	< 0·001
22 : 5	2·04	0·13	2·34*	0·11	2·18	0·12	1·99	0·15	2·10	0·14	2·15	0·17	NS
22 : 6	3·68	0·12	3·51	0·14	3·27	0·16	2·99	0·11	2·91	0·14	2·89	0·13	ND

ND, not determined.

Mean value was significantly different from that at baseline: * *P* ≤ 0·05, ** *P* ≤ 0·01.

† Ahiflower oil *v.* flax seed oil at 28 d.

‡ Statistical results are not reported because the heteroscedasticity structure could not be modelled.

**Table 5.** Polymorphonuclear cell *n*-3 PUFA (mol % of total fatty acids) at baseline and following dietary supplementation with Ahiflower or flax seed oils (Mean values with their standard errors)

	Ahiflower group						Flax group						
	Baseline		Day 14		Day 28		Baseline		Day 14		Day 28		
Fatty acid	Mean	sem	Mean	sem	Mean	sem	Mean	sem	Mean	sem	Mean	sem	Time × treatment†: *P*
18 : 3	0·11	0·01	0·18**	0·01	0·20**	0·01	0·08	0·01	0·20**	0·01	0·21**	0·01	NS
18 : 4	0·00	0·02	0·00§	0·03	0·00§	0·02	0·00	0·03	0·00§	0·05	0·00§	0·03	§
20 : 4	0·06	0·02	0·28	0·02	0·31**	0·01	0·07	0·02	0·11	0·04	0·11	0·04	< 0·001‡
20 : 5	0·14	0·02	0·23**	0·02	0·24**	0·03	0·16	0·02	0·19*	0·01	0·19*	0·02	0·01
22 : 5	1·74	0·10	1·99	0·08	1·95	0·13	1·65	0·10	1·84	0·07	1·87	0·13	NS
22 : 6	0·56	0·09	0·49	0·04	0·48	0·13	0·45	0·06	0·44	0·09	0·42	0·10	ND

ND, not determined.

Mean value was significantly different from that at baseline: * *P* ≤ 0·05, ** *P* ≤ 0·01.

† Ahiflower oil *v.* flax seed oil at 28 d.

‡ This *P* value is not associated with a time × treatment effect, it is rather associated with the effect of oil on the difference between values at baseline and at 28 d.

§ Statistical results are not reported because the heteroscedasticity structure could not be modelled.

**Table 6.** Mononuclear cells *n*-3 PUFA (mol % of total fatty acids) at baseline and following dietary supplementation with Ahiflower or flax seed oils (Mean values with their standard errors)

	Ahiflower group						Flax group						
	Baseline		Day 14		Day 28		Baseline		Day 14		Day 28		
Fatty acid	Mean	sem	Mean	sem	Mean	sem	Mean	sem	Mean	sem	Mean	sem	Time × treatment†: *P*
18 : 3	0·08	0·01	0·21**	0·01	0·23**	0·01	0·09	0·01	0·17**	0·01	0·27**	0·01	NS
18 : 4	0·00	0·04	0·03§	0·04	0·02§	0·03	0·00	0·04	0·00§	0·09	0·00§	0·05	§
20 : 4	0·03	0·05	0·21	0·02	0·23**	0·01	0·05	0·03	0·08	0·10	0·07	0·03	< 0·001‡
20 : 5	0·20	0·02	0·42**	0·02	0·48**	0·02	0·20	0·01	0·27**	0·02	0·27**	0·02	< 0·001
22 : 5	1·59	0·15	2·20**	0·10	2·44**	0·13	1·86	0·10	1·91*	0·15	2·05*	0·10	0·004
22 : 6	1·36	0·09	1·26	0·10	1·30	0·08	1·45	0·07	1·12	0·09	1·20	0·08	ND

ND, not determined.

Mean value was significantly different from that at baseline: * *P* ≤ 0·05, ** *P* ≤ 0·01.

† Ahiflower oil *v.* flax seed oil at 28 d.

‡ This *P* value is not associated with a time × treatment effect, it is rather associated with the effect of oil on the difference between values at baseline and at 28 d.

§ Statistical results are not reported because the heteroscedasticity structure could not be modelled.

#### Stearidonic acid (18 : 4*n*-3) and 8,11,14,17-eicosatetraenoic acid (20 : 4*n*-3)

SDA was undetectable in most subjects at baseline, and its accrual in plasma and in circulating cell populations was minor at 28 d in both dietary groups. With respect to ETA, this PUFA was also undetectable in plasma at baseline in most subjects. In the Ahiflower group, ETA as a percentage of total fatty acids increased significantly in plasma, in PMN and in mononuclear cells between baseline and day 28 (*P* < 0·01) and this was significantly different from the flax group (time × treatment interaction).

#### EPA (20 : 5*n*-3)

The concentrations of EPA in plasma were similar in both groups at baseline. Following 28 d of supplementation with Ahiflower oil, the concentration of plasma EPA increased 3-fold to 1·34 % of total fatty acids (*P* < 0·001), whereas the increase was less pronounced in the flax group with a less than 2-fold increase from baseline to a mean value of 0·76 % of total fatty acids (*P* < 0·001). The increase was significantly greater in the Ahiflower group than in the flax group at both 14 and 28 d (time × treatment interaction; *P* < 0·001). Additionally, after 28 d of supplementation, increases from baseline in cellular EPA were measured in both the Ahiflower and flax groups in erythrocytes, PMN and mononuclear cells. Again, the increases were significantly greater in the Ahiflower group than in the flax group in all circulating cells (time × treatment interactions at day 14, erythrocytes *P* = 0·006, PMN trended *P* = 0·07, mononuclear cells *P* = 0·001; at day 28, erythrocytes *P* < 0·001, PMN *P* = 0·01, mononuclear cells *P* < 0·001).

**Fig. 3.**
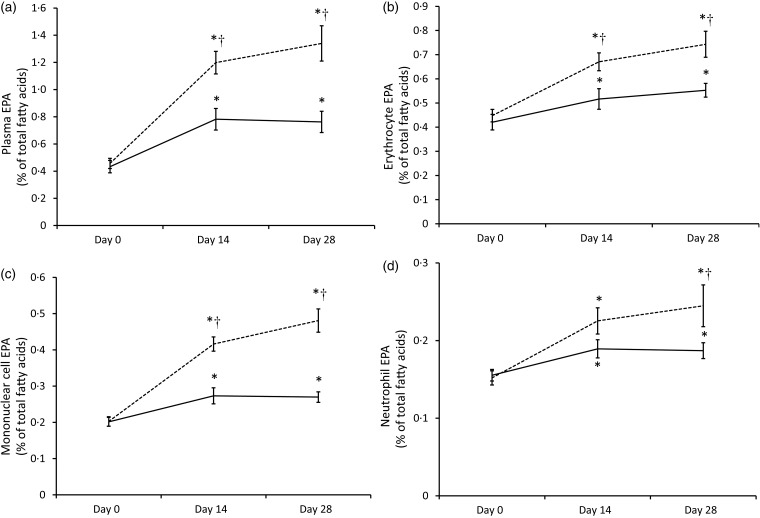
EPA content of plasma (a) and blood cell populations (erythrocytes (b); mononuclear cells (c); neutrophils (d)) expressed as a percentage of total fatty acids in Ahiflower (---) and flax (––) groups at baseline and following 14 and 28 d of supplementation. Values are means, with standard errors represented by vertical bars. * Mean value was significantly different from that at baseline (*P* ≤ 0·05). † Mean value was significantly different from that of the flax group (time × treatment interaction; *P* ≤ 0·05).

In order to illustrate the changes in EPA content over time, plasma EPA content in plasma and in each cell type as a percentage of total fatty acids is shown in Fig. 3. The most important increase in tissue EPA levels in both groups occurred during the first 14-d period. In the Ahiflower group, the EPA content continued to increase over the second 14-d period though at a slower rate of accrual, while the rate of increase in the flax group tended to level off between days 14 and 28.

#### Docosapentaenoic acid (22 : 5*n*-3)

Baseline concentrations of DPA in plasma were similar in both groups. Following 14 and 28 d of supplementation with Ahiflower oil, plasma DPA increased significantly from baseline to 0·82 % of total fatty acids (*P* < 0·01) and 0·81 % (*P* < 0·001), respectively, while DPA concentrations in the flax group increased to 0·75 % after 28 d, (*P* < 0·05). After 14 and 28 d of supplementation DPA as a percentage of total fatty acids also increased significantly compared with baseline in both the Ahiflower and flax groups in mononuclear cells. All of these increases were significantly greater in the Ahiflower group compared with the flax group (time × treatment interactions, *P* ≤ 0·05) except after 14 d in mononuclear cells where the percentage of DPA only tended to be higher in the Ahiflower group (time × treatment interaction, *P* = 0·09).

DPA did not increase in erythrocytes in the flax group after 14 d (*P* = 0·2) or 28 d (*P* = 0·3), while it increased compared with baseline in the Ahiflower group after 14 d (*P* = 0·04) and tended to increase after 28 d (*P* = 0·09). DPA tended to increase in both the Ahiflower and flax groups in PMN after 14 d (Ahiflower: *P* = 0·1, flax: *P* = 0·1) and 28 d (Ahiflower: *P* = 0·09, flax: *P* = 0·06), but these trends were similar in both groups (no time × treatment interactions).

#### Dihomo-γ-linolenic acid (20 : 3*n*-6)

Ahiflower oil contains γ-linolenic acid (GLA; 18 : 3*n*-6) that can be elongated to its 20-carbon metabolite DGLA, which possesses anti-inflammatory properties^(^^50^^)^. Table 7 shows the content of DGLA as a percentage of total fatty acids in plasma and in each cell fraction measured. The DGLA content increased significantly at day 14 and day 28 compared with baseline in all measured tissues in the Ahiflower group (except in erythrocytes at day 14), and these increases were significant compared with the flax group (time × treatment interactions) except for erythrocytes at day 14. In fact, after 28 d, the DGLA content decreased in plasma, erythrocytes and mononuclear cells and remained unchanged in PMN following supplementation with flax oil, possibly because of competition from ALA for incorporation in tissue phospholipids in the absence of supplementation with dietary GLA in that group.

**Table 7.** Dihomo-γ-linolenic acid (mol % of total fatty acids) in plasma and circulating cells at baseline and following dietary supplementation with Ahiflower or flax seed oils (Mean values with their standard errors)

	Ahiflower group						Flax group						
	Baseline		Day 14		Day 28		Baseline		Day 14		Day 28		
	Mean	sem	Mean	sem	Mean	sem	Mean	sem	Mean	sem	Mean	sem	Time × treatment†: *P*
Plasma	1·68	0·03	1·78*	0·03	1·88**	0·03	1·78	0·03	1·61**	0·03	1·50**	0·03	< 0·001
Erythrocytes	1·61	0·02	1·64	0·01	1·66*	0·01	1·80	0·01	1·68*	0·01	1·58*	0·01	< 0·001
Polymorphonuclear cells	1·68	0·04	2·06**	0·04	2·15**	0·04	1·73	0·03	1·61	0·04	1·56	0·05	< 0·001
Mononuclear cells	1·58	0·03	1·88**	0·03	1·94**	0·02	1·87	0·02	1·70	0·04	1·53**	0·03	< 0·001

Mean value was significantly different from that at baseline: * *P* ≤ 0·05, ** *P* ≤ 0·01.

† Ahiflower oil *v.* flax seed oil at 28 d.

## Discussion

This trial is the first documented study in which human subjects consumed Ahiflower oil, an oil extracted from the seeds of *B. arvensis*. Preclinical feeding studies in mice showed no adverse effects of this oil^(^^51^^)^. The present phase I clinical trial focused on measuring safety parameters, as well as efficacy in the form of enrichment of tissues with *n*-3 PUFA. Overall, no safety concerns were revealed following the consumption of 10 ml per d of Ahiflower oil formulation for a period of 4 weeks, with no clinically relevant changes in blood chemistry or haematology values, and no adverse findings in urinalyses during the course of the study. Similarly, the number of AE and AR was not different from that of subjects consuming flax seed oil and all were mild in nature.

This was a comparator-controlled trial in which Ahiflower oil was compared with flax seed oil, a commonly consumed plant-based oil and one of richest sources of dietary ALA. Overall, Ahiflower oil proved to be significantly more effective than flax seed oil at increasing the ETA, EPA and DPA content of plasma and blood cell types. Other dietary oils have been described that contain SDA, including echium oil (12–14 % SDA) and a genetically modified soyabean oil called SDA soybean oil (20–30 % SDA). In human clinical trials these oils were effective at increasing erythrocyte^(^^18^^,^^31^^–^^33^^,^^38^^,^^41^^)^, mononuclear cell^(^^34^^,^^39^^)^, neutrophil^(^^36^^)^ and plasma^(^^18^^,^^34^^,^^36^^–^^38^^,^^40^^)^ EPA and DPA content compared with baseline levels or compared with placebo controls that lack *n*-3 PUFA. However, the efficacy of SDA-containing oil was never directly compared with that of oils whose sole source of *n*-3 PUFA is ALA, like flax oil. Dittrich *et al.* recently reported hypertriacylglycerolaemic subjects consuming echium or flax seed oils; however, no statistical comparisons of tissue enrichment with *n*-3 PUFA were reported between dietary groups, therefore the extent to which SDA oils may be more efficacious than ALA oils was not determined^(^^38^^)^. The present study clearly shows that after 2–4 weeks of dietary supplementation, Ahiflower oil is significantly more effective at enriching tissues with *n*-3 PUFA elongation and desaturation metabolites of SDA, with the exception of DHA, and this was shown in three different cellular blood fractions as well as in plasma. Additionally, since Ahiflower oil contains significant quantities of ALA, tissues were also significantly enriched in this fatty acid, but to an extent that was not significantly different than that measured in subjects consuming flax seed oil.

The enhanced ability to enrich tissues in longer-chain *n*-3 PUFA with dietary Ahiflower oil compared with flax seed oil was noteworthy since both oils contained similar amounts of total *n*-3 PUFA. It is well established that the consumption of oil containing ALA results in the enrichment of tissues with ALA itself, but with smaller enrichment of tissues with EPA or DPA, largely attributed to the poor conversion of ALA to SDA as a result of limited Δ−6 desaturase activity in human tissues^(^^20^^)^. The present trial shows that the replacement of approximately 30 % of the dietary ALA with SDA, achieved by consuming Ahiflower oil instead of flax seed oil, significantly affected tissue long-chain *n*-3 PUFA, confirming that SDA is more effective at enriching tissues with elongation and desaturation products than dietary ALA. These results are also in accord with a previous study where the consumption of up to 1·5 g/d of pure SDA ethyl esters significantly increased plasma and erythrocyte EPA and DPA levels, whereas increases in long-chain *n*-3 PUFA content were not significant following the consumption of ALA ethyl esters^(^^18^^)^. However, the authors did not report whether the enrichment of tissues following the consumption of SDA ethyl esters was significantly different from that measured with dietary ALA ethyl esters.

The consumption of both Ahiflower oil and flax seed oil resulted in the greatest changes in *n*-3 PUFA content of tissues during the first 2 weeks of supplementation, although the accrual of longer-chain metabolites continued through the next 2-week period for the Ahiflower group. It can be assumed from the time–response curves for EPA (Fig. 3) that enrichment in tissues would probably have continued beyond the 4-week supplementation period. However, it is clear from the present study that significant tissue enrichment is measurable after 14 d of supplementation. Importantly, significant increases in long-chain *n*-3 PUFA were measured in leucocytes that can synthesise lipid mediators involved in both the effector and resolution phases of inflammation^(^^4^^,^^52^^)^.

Previous studies have shown that SDA-containing oils are less effective than fish oils at enriching tissues with EPA and DPA and do not make an impact on DHA levels^(^^18^^,^^31^^–^^33^^)^. In fact, when compared directly, dietary SDA was shown to be approximately three to four times less efficient at enriching erythrocytes with EPA than following the consumption of preformed EPA^(^^18^^,^^31^^–^^33^^)^. The present study is consistent with other studies that show that dietary SDA does not make an impact on tissue DHA levels, as tissue enrichment with DHA probably requires the consumption of preformed DHA. Therefore, SDA-rich plant oils cannot be considered a replacement for fish oils because dietary DHA is a precursor to several pro-resolving anti-inflammatory compounds and is important for a number of health outcomes including cognitive and visual development and function^(^^52^^–^^54^^)^. Nevertheless, the present study adds to the understanding of the impact of dietary SDA-containing oils on tissue *n*-3 PUFA by showing that Ahiflower oil is significantly more effective than ALA-containing oils at enriching tissues with longer-chain *n*-3 fatty acids like EPA and DPA. Compliance bias that is inherent in studies investigating fish oil efficacy was absent in the present study since palatability is similar between Ahiflower oil and flax seed oil.

The ability to enrich tissues with EPA and DPA following the consumption of Ahiflower oil is possibly of clinical significance since plasma long-chain *n*-3 PUFA concentrations have been inversely associated with the incidence of a number of cardiovascular outcomes in healthy cohorts from prospective studies^(^^55^^–^^58^^)^. Although the potential impact of the consumption of Ahiflower oil on cardiovascular outcomes has yet to be determined, the present study suggests that its consumption may be beneficial. Indeed, in the Japan EPA Lipid Intervention Study the consumption of pure EPA increased plasma EPA and DPA content and significantly decreased the incidence of major cardiovascular events without any impact on plasma DHA content^(^^59^^,^^60^^)^. Additionally, prospective studies have shown that the incidence of congestive heart failure and of sudden death from cardiac causes are significantly reduced when plasma EPA or when plasma 20- and 22-carbon *n*-3 PUFA content are increased by as little as 1·5-fold^(^^55^^,^^57^^)^, levels of enrichment that were achieved in the present study.

While the increase in plasma and cellular ALA was similar following the consumption of both investigational oils, the health impact of tissue enrichment with ALA is not as well established as that with long-chain *n*-3 PUFA^(^^20^^–^^22^^)^, although health benefits of ALA-containing oils in humans are beginning to emerge^(^^23^^,^^24^^)^. Similarly, the impact of enriching tissues with ETA is not known, mainly because it has not been widely investigated, as it does not appear to appreciably accumulate in tissues unless subjects consume its direct precursor SDA. An investigation of its biological properties, including those of its lipoxygenase- or cyclo-oxygenase-derived eicosanoids^(^^61^^,^^62^^)^ may begin to shed light on the potential importance of this poorly studied *n*-3 PUFA. In platelets, ETA itself inhibits cyclo-oxyenase activity as strongly as EPA^(^^62^^)^, and is a substrate for cyclo-oxygenase and platelet 12-lipoxygenase^(^^61^^)^. The fact that this fatty acid was detected in mononuclear cells and neutrophils suggests that it may be available as a substrate for eicosanoid biosynthesis by inflammatory cells.

Ahiflower oil is also a source of the *n*-6 PUFA GLA. Previous studies, including clinical trials, have shown that GLA possesses anti-inflammatory properties measured as decreases in eicosanoid biosynthesis^(^^46^^,^^50^^,^^63^^)^ and amelioration of symptoms associated with rheumatoid arthritis^(^^64^^)^. These biological effects have been largely attributable to the conversion of GLA to its elongation product DGLA. In the present study, the consumption of Ahiflower oil was associated with a significant increase in plasma and leucocyte DGLA content, indicating a possible health benefit in addition to its impact on tissue *n*-3 PUFA concentrations.

As the richest natural source of SDA, Ahiflower is the most effective non-genetically modified plant seed oil-based source of *n*-3 PUFA described to date. This is important because agriculturally produced oils are renewable sources of dietary *n*-3 PUFA as opposed to fish oils, whose sustainability is questionable^(^^13^^–^^15^^)^. Given the ability to enrich tissues in EPA and other long-chain *n*-3 PUFA following its consumption, Ahiflower oil represents an attractive alternative to current plant-sourced ALA-rich oils as a dietary source of *n*-3 PUFA.

## Supplementary material

To view supplementary material for this article, please visit http://dx.doi.org/jns.2015.34
